# Foreleg Transcriptomic Analysis of the Chemosensory Gene Families in *Plagiodera versicolora* (Coleoptera: Chrysomelidae)

**DOI:** 10.3390/insects13090763

**Published:** 2022-08-24

**Authors:** Zheran Wu, Na Tong, Yang Li, Jinmeng Guo, Min Lu, Xiaolong Liu

**Affiliations:** 1 State Key Laboratory of Biocatalysis and Enzyme Engineering, School of Life Sciences, Hubei University, Wuhan 430062, China; 2 Key Laboratory of Integrated Management of Crop Disease and Pests, Ministry of Education/Department of Entomology, Nanjing Agricultural University, Nanjing 210095, China

**Keywords:** *Plagiodera versicolora*, forelegs transcriptome, odorant binding proteins, chemosensory proteins

## Abstract

**Simple Summary:**

The chemosensory system plays an important role in insect behavior, such as foraging, mating, host-seeking, oviposition, and so on. In contrast with the transcriptome of the antennae, the transcriptome of the forelegs studied in Coleoptera are much less understood. In this study, we performed a transcriptome analysis of adult forelegs in *Plagiodera versicolora* and 53 candidate chemosensory genes encoding 4 chemosensory proteins (CSPs), 19 odorant binding proteins (OBPs), 10 odorant receptors (ORs), 10 gustatory receptors (GRs), 6 ionotropic receptors (IRs), and 4 sensory neuron membrane proteins (SNMPs) were identified. Then, the expression of the chemosensory genes was examined using real-time quantitative PCR. These results will provide valuable information to help explore the chemoreception mechanism in *P. versicolora.*

**Abstract:**

*Plagiodera versicolora* (Coleoptera: Chrysomelidae) is a worldwide leaf-eating forest pest in salicaceous trees. The forelegs play important roles in the chemoreception of insects. In this study, we conducted a transcriptome analysis of adult forelegs in *P. versicolora* and identified a total of 53 candidate chemosensory genes encoding 4 chemosensory proteins (CSPs), 19 odorant binding proteins (OBPs), 10 odorant receptors (ORs), 10 gustatory receptors (GRs), 6 ionotropic receptors (IRs), and 4 sensory neuron membrane proteins (SNMPs). Compared with the previous antennae transcriptome data, 1 CSP, 4 OBPs, 1 OR, 3 IRs, and 4 GRs were newly identified in the forelegs. Subsequently, the tissue expression profiles of 10 *P. versicolora* chemosensory genes were performed by real-time quantitative PCR. The results showed that PverOBP25, PverOBP27, and PverCSP6 were highly expressed in the antennae of both sexes. PverCSP11 and PverIR9 are predominately expressed in the forelegs than in the antennae. In addition, the expression levels of PverGR15 in female antennae and forelegs were significantly higher than those in the male antennae, implying that it may be involved in some female-specific behaviors such as oviposition site seeking. This work would greatly further the understanding of the chemoreception mechanism in *P. versicolora.*

## 1. Introduction

Insects are the most diverse group of animals on Earth [1]. Communication with the environment and conspecific individuals through chemical signals is an essential process for the survival of insects [2]. Chemoreception is responsible for recognizing the external chemical signals that influence the behavior of insects [1]. Several peripheral olfactory proteins are involved in chemoreception, including odorant receptors (ORs) [3,4], gustatory receptors (GRs) [4], ionotropic receptors (IRs) [5], odorant binding proteins (OBPs) [6], chemosensory proteins (CSPs) [7], and sensory neuron membrane proteins (SNMPs) [8].

OBPs and CSPs play an important role in chemical signal perception [7]. OBPs and CSPs are small soluble polypeptides in insects. OBPs generally consist of chains of about 150 amino acids, and CSPs are smaller than OBPs, with 100–120 residues [6]. They generally have a signal peptide sequence of approximately 20 amino acids at the N terminus. OBPs presents the common motif of the six α-helices with six cysteines forming disulphide bridges, while CSPs present a motif of four conserved cysteines linked by disulphide bridges between neighboring residues [9].

The origin of GRs probably traces back to the origin of the arthropods, and presumably evolved early from ORs [10,11]. GRs are ligand-gated cation channels, and encode seven transmembrane domain proteins with an intracellular N terminus and an extracellular C terminus [12,13]. Insect GRs are mainly expressed in gustatory receptor neurons (GRNs) of the gustatory organs (found on the antennae, proboscis, maxillary palps, legs, and wings) [14,15,16]. GRs generally detect non-volatile chemicals such as sugars, amino acids, and bitter-tasting compounds, as well as carbon dioxide (CO_2_) and pheromones [17].

IRs were initially found in the *Drosophila melanogaster* genome using bioinformatic and expression screening [5]. Insect IRs are a novel family of chemosensory receptors that are related to ionotropic glutamate receptors (iGluRs), and act as ligand-based ion channels [18,19]. Similar to ORs, the specific antennal IRs form heteromeric complexes with one or more co-receptors (IR8a, IR25a, or IR76b) to perform their physiological functions [5,19,20,21,22]. In general, the IRs protein family may be involved in detecting diverse chemical ligands to mediate both intercellular communication and environmental chemical sensing [23,24,25].

Plant chemicals can be detected by the chemoreceptors on the tarsi when phytophagous insect tarsi directly contact the plant [26]. Many studies have found that chemosensilla on insect legs play a central role in chemosensory detection and in the selection of host plants. The contact chemoreceptors involved in the perception of mustard oil glucosides are located on the tarsi of the legs in *Pieris brassicae* [27]. Abundant taste and mechanosensory bristles are located on the legs, proboscis, and the anterior wing margins in *D. melanogaster* [28,29]. Fourteen gustatory trichoid chemosensilla in the fifth tarsomere of the prothoracic legs of female adult *Helicoverpa armigera* and the proboscis extension reflex (PER) was induced when the tarsi of their legs was touched with sugar [30].

With the development of the transcriptome, chemosensory genes were identified from large amounts of insects in Coleoptera [31,32]. The willow leaf beetle, *Plagiodera versicolora* (Laicharting; Coleoptera: Chrysomelidae), is a specialist herbivore on salicaceous trees [33]. However, there is a blank of identified chemosensory gene families in the forelegs of *P. versicolora*. In a previous study, 40 ORs, 7 IRs, 13 GRs, 10 CSPs, 24 OBPs, and 4 SNMPs were identified from the antennae transcriptome [34]. In this study, we conducted a transcriptomic analysis of the forelegs in *P. versicolora* and identified 53 candidate chemosensory genes, including 10 ORs, 19 OBPs, 4 CSPs, 6 IRs, 10 GRs, and 4 SNMPs. Then, we constructed phylogenetic trees and explored the evolutionary relationships of these chemosensory genes with other coleopteran insects, as well as those chemosensory genes already identified from the antennal transcriptome. Furthermore, real-time quantitative PCR experiments (RT-qPCR) were conducted to investigate the tissue expression pattern in both sexes. These results may provide insight into the molecular mechanism of chemosensory genes in *P. versicolora*.

## 2. Materials and Methods

### 2.1. Insect Rearing and Tissue Collection

The larvae and adults of *P. versicolora* were captured from Sha Lake Park in Wuhan, Hubei Province, and reared in the lab under the following conditions of 28 ± 1 °C, a photoperiod of 12-h light/12-h dark, and 70% ± 5% relative humidity. Larvae and adults were fed with fresh leave of willows. The 300 male and female forelegs tissues were excised from 2-day-old adults, respectively. After collection, the tissues were immediately frozen in liquid nitrogen and stored at −80 °C until RNA extraction.

### 2.2. RNA Extraction, cDNA Library Preparation, and Sequencing

The total RNA was extracted from male and female forelegs with the TRIZOL reagent (Invitrogen, Carlsbad, CA, USA) following the manufacturer’s protocol. The RNA concentration was determined using NanoDrop2000 and the RNA integrity number was checked with Agilent2100 (OD260/280 ≥ 1.8 and OD260/230 ≥ 1.0). Six RNA samples of male and female forelegs were used to construct the complementary DNA (cDNA) library. The mRNA was enriched by base pairing in magnetic beads with oligo d(T) and polyA. Then, the mRNA was fragmented with a fragmentation buffer, and a small fragment of about 300 bp was isolated by magnetic bead screening. After this, six base random primers were added to reverse synthesize the one strand cDNA used in the mRNA as a template, and then two strand cDNA was synthetized and formed a stable double strand structure. The libraries were sequenced using the Illumina Novaseq 6000 and performed at Shanghai Majorbio Bio-pharm Technology Co., Ltd. (Shanghai, China).

### 2.3. Assembly and Functional Annotation

The connector sequences in the raw reads and the reads without inserted fragments were removed. Low-quality reads, including reads containing > 10% N and median quality value (Q) ≤ 25, were also removed to generate clean reads. All clean-read datasets were used for de novo assembly by Trinity (https://github.com/trinityrnaseq/trinityrnaseq/wiki, accessed on 10 December 2021). Then, the trinity outputs were estimated by TransRate (http://hibberdlab.com/transrate/, accessed on 10 December 2021) and redundant sequences were removed by using CD-HIT (http://weizhongli-lab.org/cd-hit/, accessed on 10 December 2021).Unigenes were functionally annotated by BLAST-searching the databases against the NCBI non-redundant protein sequences (NR), Pfam, Clusters of Orthologous Groups of Proteins (COG), Kyoto Encyclopedia of Genes and Genomes (KEGG), Gene Ontology (GO), and Swiss-Prot databases.

### 2.4. Sequence Analysis and Phylogenetic Tree Construction

The complete coding region was determined by using ORF finder (http://www.ncbi.nlm.nih.gov/gorf/gorf.html, accessed on 20 June 2022). The signal peptides of the putative OBPs and CSPs were predicted using the SignalP 5.0 server (http://www.cbs.dtu.dk/services/SignalP/, accessed on 20 June 2022). The transmembrane domains (TMDs) of ORs, GRs, IRs, and SNMPs were predicted using TMHMM Server v.2.0 (http://www.cbs.dtu. dk/services/TMHMM/, accessed on 12 June 2022).

A set of chemosensory genes from *P. versicolora* (Chrysomelidae) and different insect species were obtained, including five Coleoptera from different families, *Colaphellus bowringi* (Chrysomelidae) [35], *Aethina tumida* (Nitidulidae Latreille) [36], *Dastarcus helophoroides* (Colydiidae) [37], *Monochamus alternatus* (Cerambycidae) [37], and *Anoplophora chinensis* (Cerambycidae) [38], to construct OBP, CSP, and GR phylogenetic trees. In addition, the GR amino acid sequences that have been functionally identified, including three Lepidoptera, *Plutella xylostella* [39], *H. armigera* [40,41,42], *Bombyx mori* [43,44,45], and three Diptera, *D. melanogaster* [46,47,48,49], *Aedes aegypti* [13], and *Anopheles gambiae* [50], were used to construct a phylogenetic tree. The phylogenetic trees were conducted in MEGA6 using the neighbor-joining method with a bootstrap test (1000 replicates). The phylogenetic trees were viewed and edited using FigTree v. 1.4.3 (http://tree.bio.ed.ac.uk/software/figtree/, accessed on 25 June 2022).

### 2.5. Expression Analysis by Real-Time Quantitative PCR

The expression patterns of the chemosensory genes in different tissues, including the antennae, forelegs, and middle and hind legs in both sexes were analyzed by RT-qPCR. Specific primer pairs were designed by Primer 5 (Table S1). The total RNA from the analyzed tissues was extracted as described above. The synthesis of the cDNA, RT-qPCR conditions, and reaction program were same as those previously described [34]. The *RPS18* gene [51] was used as the reference gene. Three biological duplications of each gene were performed in our experiments. The relative gene expression level was quantified using the comparative 2^−ΔΔCT^ method [52]. One-way ANOVA was used to analyze the relative expression levels of these genes using SPSS 22.0 software (IBM, Armonk, NY, USA). 

## 3. Results

### 3.1. Transcriptome Analysis and Assembly

The transcriptome sequencing of male and female forelegs was performed to identify the chemosensory genes of *P. versicolora*. By de novo assembly, approximately 69.57, 60.35, and 65.49 million raw reads of female forelegs, and 70.60, 71.97, and 66.73 million raw reads of male forelegs were obtained, respectively. After filtering, 68.80, 59.55, and 64.78 million clean reads of female forelegs, and 69.87, 71.13, and 65.94 million clean reads of male forelegs were generated, respectively. The Busco analysis confirmed the completeness of the transcriptome (C: 95.7% [S: 93.8%; D: 1.9%]). A final transcript dataset with 28,977 unigenes was obtained, with a mean length of 1229 bp and N50 of 2644 bp. The analysis of unigene length distribution indicated that 9214 unigenes (31.8%) were greater than 1000 bp, 4889 unigenes (17.52%) were 500 to 1000 bp, and 14,140 unigenes (50.68%) were 200 to 500 bp.

**Figure 1:**
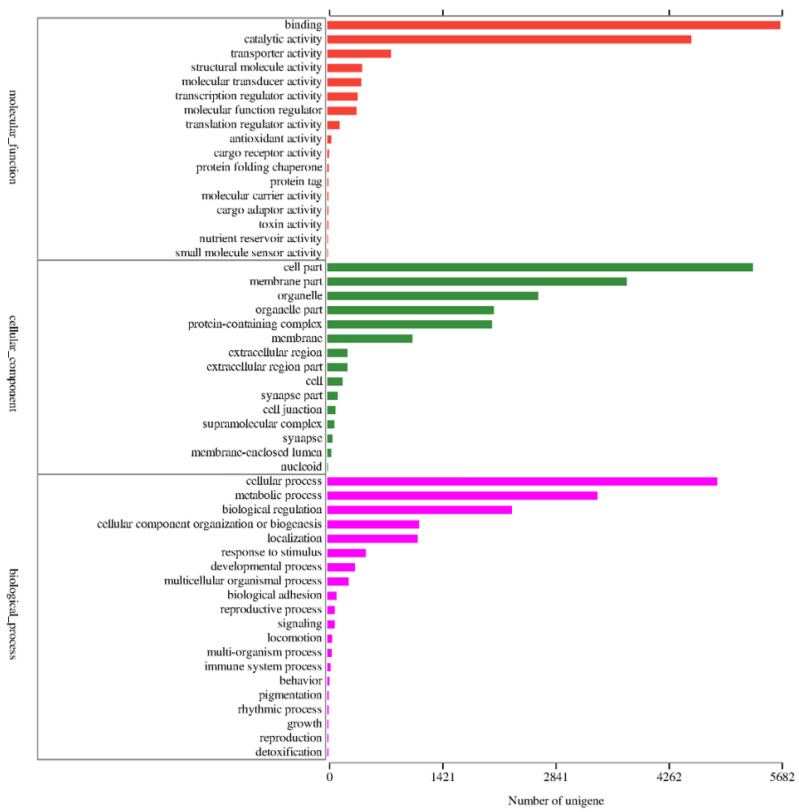
Gene ontology (GO) classification of foreleg transcriptome unigenes in *P. versicolora*.

The GO annotation analysis showed that 10,674 unigenes (37.89%) could be annotated into three functional categories: biological process, cellular component, and molecular function. The most abundant group of cellular component categories was cell part (5335 unigenes), and cellular process was the most represented in terms of biological process (4891 unigenes). In the molecular function term, binding (5682 unigenes) was the most abundant for all unigenes. The genes expressed in the forelegs were mostly associated with binding and transporter activities (793), indicating that unigenes might take part in chemosensory behavior (Figure 1).

### 3.2. Identification of Putative OBP Genes

**Figure 2:**
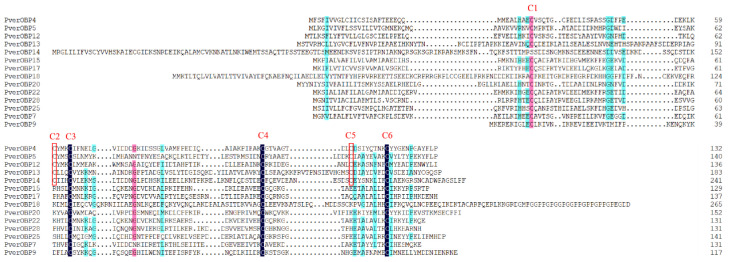
Alignment of the full-length amino acid sequence of Classic OBPs and Minus-C OBPs identified in this study. Conserved cysteine residues are marked with a red box (C2 and C5), or black (C3, C4, and C6) and pink (C1) background.

**Figure 3:**
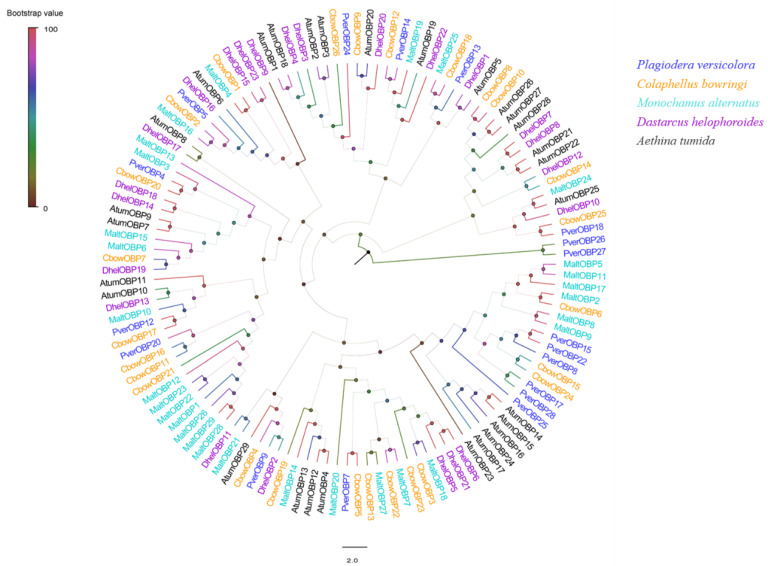
Phylogenetic analysis of the OBPs (odorant-binding proteins) from five insect species: *P. versicolora* (Pver), *C. bowringi* (Cbow), *M. alternatus* (Malt), *D. helophoroides* (Dhel), and *A. tumida* (Atum). The amino acids sequences without signal peptides were used. The *P. versicolora* genes are shown in blue.

In this study, 19 candidate OBPs (four newly compared with antennae) were identified from the foreleg’s transcriptome data of *P. versicolora*. The complete ORF was detected in 14 OBPs, with lengths ranging from 129 to 266 amino acids. Among them, 14 OBPs had a signal peptide sequence (Table S2). The further alignment of these 14 amino acid sequences showed that four OBPs belonged to the classical OBP subgroup with the six conserved cysteine (C) residues. Nine OBPs belonged to the minus-C OBP subgroup with four conserved C residues (Figure 2). The phylogenetic analysis showed that PverOBP4 and PverOBP12 clustered with MaltOBP3 and MaltOBP10, two proteins that bound plant-related compounds (Figure 3).

### 3.3. Identification of Putative CSP Genes

**Figure 4:**
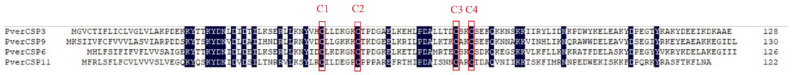
Alignment of the full-length amino acid sequence of CSPs identified in this study. Conserved cysteine residues are marked with a red box.

**Figure 5:**
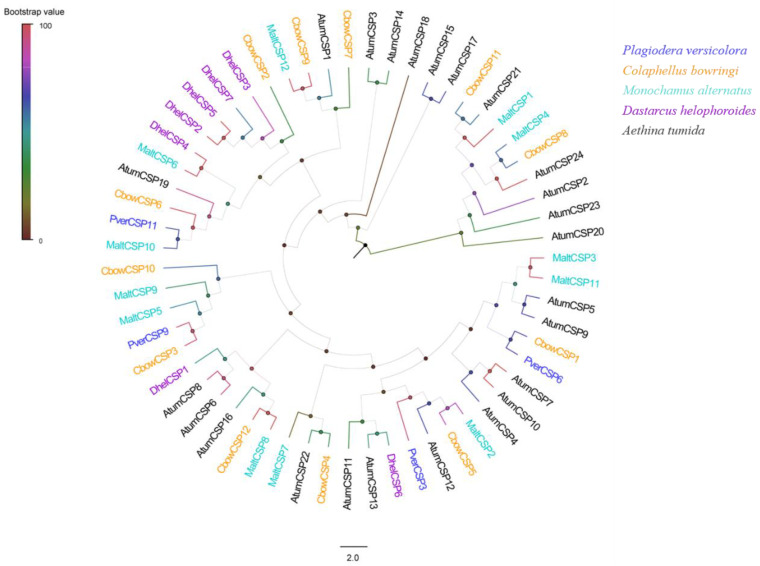
Phylogenetic analysis of the CSPs (chemosensory proteins) from five insect species: *P. versicolora* (Pver), *C. bowringi* (Cbow), *M. alternatus* (Malt), *D. helophoroides* (Dhel), and *A. tumida* (Atum). The *P. versicolora* genes are shown in blue.

Four putative CSPs (one newly compared with antennae) were identified by analyzing the transcriptome data of *P. versicolora*. The sequence analysis revealed that all four putative CSPs had a full length ORFs, with a length from 122 to 131 amino acids and a predicted signal peptide (Table S2). The further alignment of the amino acid sequences showed that four CSPs had the six conserved C residues (Figure 4). The CSPs phylogenetic tree indicated that PverCSP9 was in the same branch as MaltCSP5, which has been functionally characterized (Figure 5).

### 3.4. Identification of Putative OR Genes

A total of 10 candidate ORs (one newly compared with antennae) were identified in the foreleg transcriptome of *P. versicolora* through a keyword search of the BLASTx annotation. Sequence analysis revealed that only one OR was predicted to have a full length ORF of 417 amino acids, and contained eight TMDs (Table S3).

### 3.5. Identification of Putative GR Genes

**Figure 6:**
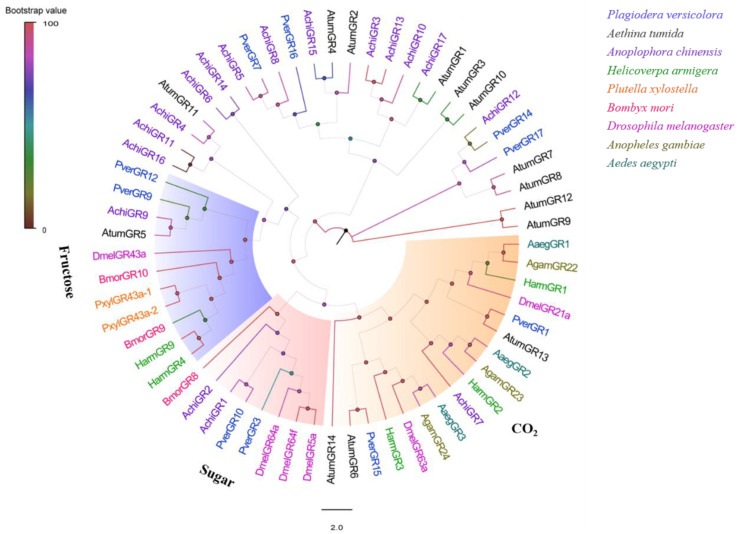
Phylogenetic analysis of the GRs (gustatory receptors) from nine insect species: *P. versicolora* (Pver), *A. tumida* (Atum), *A. gambiae* (Agam), *A. chinensis* (Achi), *H. armigera* (Harm), *P. xylostella* (Pxyl), *D. melanogaster* (Dmel), *B. mori* (Bmor), and *A. aegypti* (Aage). The *P. versicolora* genes are shown in blue.

A total of 10 candidate GR (four newly compared with antennae) transcripts were identified in both male and female *P. versicolora* foreleg transcriptomes. Of them, only PverGR9 and PverCR15 (newly) encoded full-length proteins (426 and 445 amino acids, respectively) with seven TMDs (Table S3). The phylogenetic analysis showed that PverGR9 and PverGR12 were clustered with fructose receptors, PverGR3 and PverGR10 were members of the sugar receptors subfamily, and PverGR1 and PverGR15 formed a clade with CO_2_ receptors (Figure 6).

### 3.6. Identification of Putative IR Genes

A total of six candidate IRs (three newly compared with antennae) were identified from *P. versicolora* transcriptomes by bioinformatics analysis according to a comparison with known insect IRs. Among these IRs, three encoded full-length proteins with 804 to 918 amino acid residues, the remaining IRs were incomplete, due to a lack of complete 5′ or 3′ terminus. Of these IRs, the transmembrane domains ranged from 0 to 4 (Table S3).

### 3.7. Identification of Putative SNMP Genes

Four putative SNMPs (same as the antennae transcriptome) were identified in our transcripts based on the BLASTx and cluster analysis results. SNMP1b and SNMP2a have full-length ORFs encoding 534 and 515 amino acids, respectively. In addition, the candidate SNMP1b and SNMP2a contained two TMDs, while SNMP1a and SNMP2a contained only one (Table S3).

### 3.8. Tissue Specific Expression Analysis of Putative Chemosensory Genes

**Figure 7:**
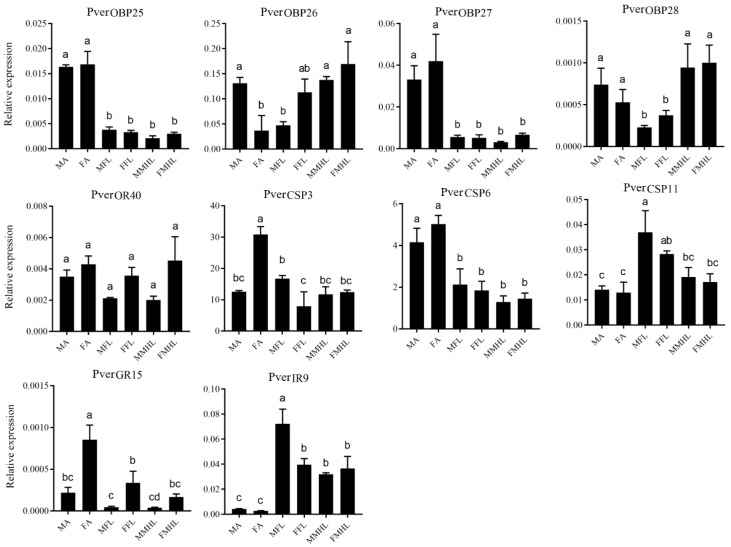
Expression levels of chemosensory genes in different tissues assessed by RT-qPCR. MA, male antennae; FA, female antennae; MFL, male forelegs; FFL, female forelegs. MMHL, male middle and hind legs; FMHL, female middle and hind legs. Error bars indicate standard error of three biological replicates. Different letters (a–c) indicate significant differences (*p* < 0.05) based on one-way ANOVA.

RT-qPCR was used to analyzed the tissue specific expression profile of 10 chemosensory genes in the antennae and legs (Figure 7). In the expression analyses, 13 candidate chemosensory genes that were newly identified in the forelegs (Figure S1) were chosen, and 8 (OBP25, OBP26, OBP27, OBP28, OR40, CSP11, GR15, and IR9) specific RT-qPCR primer were obtained. In addition, two PverCSPs (CSP3 and CSP6) were chosen that were both identified from forelegs and antennae. The results showed that PverOBP25 and PverOBP27 were highly expressed in the antennae of males and females. PverOBP28 was highly expressed in the antennae, as well as middle and hind legs than in the forelegs. The results revealed that the expression level of PverOR40 had no significant difference in antennae, forelegs, and middle and hind legs. The tissue expression profiles showed that PverCSP3 had a higher expression in female antennae and PverCSP6 in male and female antennae. In addition, PverCSP11 was highly expressed in male forelegs than in the antennae, and middle and hind legs. PverGR15 expression levels in female were significantly higher than in the male antennae and forelegs. PverIR9 has a higher expression in the forelegs, and middle and hind legs than in the antennae.

## 4. Discussion

Volatile chemicals are mainly detected by antennae in insects; while a certain number of chemosensory genes are located in the leg tarsus, current studies have mainly focused on the putative chemosensory genes in the forelegs. In order to better understand the molecular mechanism of chemosensory perception, the first step is to investigate chemosensory genes, which encode the proteins that function in odorant molecular detection. The transcriptome analysis of the forelegs in *P. versicolora* was conducted to identify the chemosensory genes. The phylogenetic analyses were carried out on these genes to examine the similarities and differences of related genes. In addition, the tissue expression profile analysis of 10 chemosensory genes was tested by RT-qPCR.

Nineteen candidate OBPs were identified through forelegs transcriptome data in *P. versicolora*. This number was more than that previously reported, which identified nine and eight OBPs in the forelegs of *A. aegypti* [53] and *Adelphocoris lineolatus* [54], and 15 OBP were identified from the assembled legs transcriptome in *Apis cerana cerana* [55]. The number of OBP family is expanded in *P. versicolora*. However, the number was less than the transcriptome data of the forelegs, and middle and hind legs of adult bugs demonstrated 20 OBP in *Apolygus lucorum* [56]. As for CSPs, four PverCSPs were obtained from the foreleg transcriptome data in *P. versicolora*, which is less than five and eight CSPs in *A. c. cerana* and *A. lucorum* from legs (including forelegs, and middle and hind legs) [55,56], respectively. More PverCSP and PverOBP genes may be identified from the transcriptome data of middle and hind legs in the future.

In the phylogenetic tree, one cluster shares high sequence identities, and usually these genes have a similar function. For example, in the OBP phylogenetic tree, AlepOBP6 in *Athetis lepigone* is clustered into the moth ABPX subfamily [57], and the function was similar to two functionally characterized OBP, BmorABPX in *B. mori* [58] and AlinOBP4 in *A. lineolatus* [59]. In our study, the result of a phylogenetic tree showed that PverOBP4, PverOBP12, and PverCSP9 clustered with MaltOBP3, MaltOBP10, and MaltCSP5 that have been functionally characterized, respectively, suggesting these PverOBPs and PverCSPs may have a similar function. The ligand-binding function of these genes needs to be investigated further. In the analysis, OBP families exhibited clades that included multiple paralogues from different species. Five PverOBPs (8, 15, 17, 22, and 28) were clustered together, suggesting that the genes of the family have undergone gene duplication. The CSP gene families shows a similar evolutionary pattern. Four PverCSPs (3, 6, 9, and 11) belong to different gene clades. Most GR families are unique and show a different species specificity. However, the CO_2_ receptors, fructose receptors, and sugar receptors are conserved.

The insect OBPs and CSPs are expressed in different tissues (antennae, legs, abdomens, wings, and so on) and are involved in the perception of chemical substances. AlucCSP2 were specifically expressed in female wings and could bind the cotton secondary metabolites [60]. MsepCSP8 was specifically expressed in the antennae and the behavioral response experiments showed it had a high binding affinity to plant volatiles [61]. MaltOBP10 was specifically expressed in the antenna of females and had high binding affinities with many volatiles from forests. PverOBP25, PverOBP27, PverCSP3, and PverCSP6 were specifically expressed in the antennae and these genes may play a role in the fundamental olfactory recognition process. PverCSP3 and PverCSP6 were specifically expressed in the antennae and may be involved in host seeking. AlucCSP3 in *A. lucorum* showed significantly higher expression levels in the forelegs than the middle and hind legs of males [56]. Similarly, PverCSP11 was the most abundantly expressed in male forelegs, and we hypothesized it would be associated with the recognition of these contact substances on host plant surfaces or courtship behavior. CSPs play multiple roles beyond chemosensation. CSP10 in *Periplaneta americana* seems to be a major extracellular matrix protein during limb regeneration [62]. The CSP gene family in *Chroistoneura fumiferana* may be involved in development, including molting [63]. CSP11 in *Bombyx mori* was involved in the recovery and synthesis of damaged skin [64]. More of the functions of these PverCSPs need to be further characterized.

Insect IRs are further classified into two sub-families: conserved “antennal IRs”, which are highly or specifically expressed in the antennae and are mainly involved in olfactory sensation, whereas the species-specific “divergent IRs” are primarily distributed in non-antennal tissues, and some of them can be responsible for the sensation of taste [65,66,67,68,69]. For instance, IR25a and IR76b act as co-receptors and are co-expressed with divergent IRs together in legs, and respond to taste in *D. melanogaster* [25,70]. The RT-qPCR results showed that PverIR9 had a higher expression in the legs than the antennae, and was the most abundantly expressed in male forelegs than in other tissues. It has been indicated that PverIR9 is related to host or mate selection, suggesting a potential role in taste sensation or other physiological processes, such as male courtship.

Ten PverGRs were obtained from transcriptomes of the forelegs, of which four GRs were newly identified, compared with the previous antennae transcriptome. The phylogenetic analysis showed that PverGR9 and PverGR12 were assigned to group fructose receptors, and PverGR3 and PverGR10 were assigned to group sugar receptors, indicating these four PverGRs might be involved in the detection of sugar. PverGR1 was obtained in the antennae of *P. versicolora*, which belongs to the GR1 subfamily in the CO_2_ receptor group [34]. In this study, PverGR15 was identified in the forelegs of *P. versicolora*, which belongs to GR3 subfamily in the CO_2_ receptors group according the tree, suggesting it might be involved in CO_2_ detection. The third CO_2_ receptor may identify it from other taste tissues, such as proboscises, labial palps, and ovipositors [71,72]. In addition, the expression levels of PverGR15 in female antennae and forelegs were significantly higher than those in males, indicating that PverGR15 may be related to some female-specific behaviors in insects such as oviposition site seeking or courtship cues. The specific functions of these GRs need to be further characterized by using, for example, a *Xenopus* oocytes combined two-electrode voltage-clamp system in vitro or RNAi technology in vivo.

## 5. Conclusions

In the present study, 53 candidate chemosensory genes (19 OBPs, 4 CSPs, 10 ORs, 6 IRs, 10 GRs, and 4 SNMPs) were identified from a transcriptome analysis of the forelegs in *P. versicolora*. Compared with the previous antennae transcriptome data, one CSP, four OBPs, one OR, three IRs, and four GRs were newly identified. The results of PR-qPCR showed that PverCSP11 and PverIR9 were predominately expressed in the forelegs than in the antennae. PverOBP25, PverOBP27, and PverCSP6 were highly expressed in the antennae of both sexes. This work would greatly promote the understanding of the olfactory system and provides helpful for functional studies of the chemoreception mechanism in *P. versicolora*.

## Data Availability

No new data were created or analyzed in this study. Data sharing is not applicable to this article.

## Supplementary Materials

The following are available online at https://www.mdpi.com/article/10.3390/insects13090763/s1. Table S1: Primers for RT-qPCR of chemosensory genes in *P. versicolora*. Table S2: The Blastx match of *P. versicolora* candidate CSP and OBP genes. Table S3: The Blastx match of *P. versicolora* candidate GR, IR, OR, and SNMP genes. File S1: The amino acid sequences of *Plagiodera versicolora* putative chemosensory genes. Figure S1: The Venn diagrams of chemosensory genes in *P. versicolora* from the transcriptome of antennae and forelegs.
