# Supplementary material for: Foreleg Transcriptomic Analysis of the Chemosensory Gene Families in Plagiodera versicolora (Coleoptera: Chrysomelidae)
Source: Insects. 2022 Aug 24;13(9):763. doi: 10.3390/insects13090763 (PMC9503008; doi:10.3390/insects13090763)
Supplement: Supplementary file 1 [file insects-13-00763-s001.zip › insects-1823238-supplementary.pdf]

Supplementary data

Table S1. Primers for RT-qPCR of chemosensory genes in *P. versicolora*.

OBP25-F	GACGAACGGTTGGCTACTT	CSP3-F	CATTTACCGGATGCCCTACTC
OBP25-R	CTCCGGGTAATACTCGTTGATG	CSP3-F	TTCGAGCTCTTTGTACCAATCA
OBP26-F	GACTTATCGTCAGATACCAAGTCG	CSP6-R	GAGAACCGAATGCAGCAAATG
OBP26-R	TCTGCATGTCGGTGAAAGAG	CSP6-F	GTTCATCAAAGGTAGCCCTCTTA
OBP27-F	AGTTGTTTCTCCATTCGATTCA	CSP11-F	GCTAGGGAATTCCGAACACATA
OBP27-R	GTCTTTGACAGGGCATTCATC	CSP11-R	TATGTTGACCTGTGCGTCTG
OBP28-F	GACGACTCAGTGGTAGAAGATG	GR15-F	CACCGTTTATCTGGTGCCTAT
OBP28-R	GTTTATGGAGACACCTGGTTAGA	GR15-R	TACCGACAGGTTCTTCCTAGT
OR40-F	GGAGCTTCTTTCCTAGCCTATTC	IR9-F	CGAAGCCTTGTCGAAACAAAC
OR40-R	CAAAGTTGGCTACAATATGCTTCA	IR9-F	GTTCAGCCACATCCTCTGATAC
q18S-F	CTTCCTCGTCGGAGCATTCT	q18S-R	GTTCGCCTTAACTGCCATCAA

Table S2. The Blastx match of *P. versicolora* candidate CSP and OBP genes

Name	ORF (aa)	Signal Peptide	Complete	Blast best hit ACC. NO. Gene Species	Evalue	Identity (%)
CSP3	128	1-17	Y	QFO46789.1| chemosensory protein [Cylas formicarius]	5e-56	68.8
CSP6	126	1-18	Y	ALR72515.1| chemosensory protein 1 [Colaphellus bowringi]	4e-52	71.2
CSP9	131	1-18	Y	ALR72517.1| chemosensory protein 3 [Colaphellus bowringi]	2e-49	67.2
CSP11	122	1-19	Y	AUF73000.1| chemosensory protein [Anoplophora chinensis]	8e-48	65.4
OBP4	132	1-17	Y	ALR72508.1| odorant binding protein 20 [Colaphellus bowringi]	9e-56	60.6
OBP5	140	1-19	Y	ALR72490.1| odorant binding protein 2 [Colaphellus bowringi]	1e-57	59.7
OBP6	135	1-18	Y	AWK23450.1| odorant-binding protein 13 [Chrysomela populi]	3e-81	94.8
OBP7	131	1-17	Y	AXO78397.1| odorant binding protein 19 [Xylotrechus quadripes]	3e-21	43.6
OBP8	68	N	N	APC94195.1| odorant-binding protein 7 [Pyrrhalta maculicollis]	8e-06	41.9
OBP9	117	N	N	ALR72492.1| odorant binding protein 4 [Colaphellus bowringi]	4e-33	58.2
OBP12	136	1-18	Y	ALR72505.1| odorant binding protein 17 [Colaphellus bowringi]	8e-70	75
OBP13	183	1-23	Y	AUF72969.1| odorant-binding protein [Anoplophora chinensis]	3e-84	70
OBP14	241	1-18	Y	ALR72500.1| odorant binding protein 12 [Colaphellus bowringi]	1e-61	47.2
OBP15	129	1-19	Y	AQY18986.1| odorant-binding protein [Galeruca daurica]	5e-26	40.6
OBP17	137	1-21	Y	ALR72503.1| odorant binding protein 15 [Colaphellus bowringi]	2e-16	40.4
OBP18	266	1-20	Y	ALR72513.1| odorant binding protein 25 [Colaphellus bowringi]	1e-85	59.6
OBP20	152	1-23	Y	ALR72504.1| odorant binding protein 16 [Colaphellus bowringi]	1e-24	36.7
OBP22	131	1-19	Y	ALR72503.1| odorant binding protein 15 [Colaphellus bowringi]	3e-25	55.2
OBP24	98	N	N	BAA88062.1| odorant binding protein-2 [Phyllopertha diversa]	9e-19	45.6
OBP25	141	1-19	Y	AQY18990.1| odorant-binding protein [Galeruca daurica]	4e-18	34.3
OBP26	92	N	N	ALR72494.1| odorant binding protein [Colaphellus bowringi]	5e-35	70.4
OBP27	86	N	N	XP_023027761.1| general odorant-binding protein 83a-like [Leptinotarsa decemlineata]	0.003	38.6
OBP28	131	1-20	Y	ALR72503.1| odorant binding protein 15 [Colaphellus bowringi]	6e-24	40.91

Table S3. The Blastx match of *P. versicolora* candidate GR, IR, OR and SNMP genes

Name	ORF (aa)	TMD	Complete	Blast best hit ACC. NO. Gene Species	Evalue	Identity (%)
GR1	119	–	N	AVH87329.1| gustatory receptor 14 [Holotrichia parallela]	5e-64	78.9
GR3	216	–	N	AUF73052.1| gustatory receptor [Anoplophora chinensis]	1e-43	41.0
GR7	304	–	N	APC94333.1| gustatory receptor 3 [Pyrrhalta aenescens]	9e-30	28.7
GR9	426	7	Y	AVN97874.1| gustatory receptor 9 [Anoplophora chinensis]	4e-80	38.2
GR10	100	–	N			
GR12	106	–	N	AVN97874.1| gustatory receptor 9 [Anoplophora chinensis]	0.019	36.7
GR14	235	–	N	APC94341.1| gustatory receptor 14 [Pyrrhalta aenescens]	1e-26	33.1
GR15	445	7	Y	RZC42344.1| gustatory and odorant receptor 24 [Asbolus verrucosus]	5e-172	59.8
GR16	101	–	N	APC94248.1| gustatory receptor 3 [Pyrrhalta maculicollis]	6e-10	31.8
GR17	139	–	N	APC94340.1| gustatory receptor 13 [Pyrrhalta aenescens]	2e-08	37.3

IR2	502	–	N	AKC58589.1|	ionotropic receptor 75q [Anomala corpulenta]	4e-95	37.5
IR4	827	3	Y	ALR72538.1|	ionotropic receptor 8a [Colaphellus bowringi]	0.0	68.4
IR5	804	2	Y	ALR72535.1|	ionotropic receptor IR6 [Colaphellus bowringi]	0.0	82.7
IR8	259	–	N	APC94262.1|	ionotropic receptor 4 [Pyrrhalta maculicollis]	3e-27	50.4
IR9	918	4	Y	ANQ46493.1|	ionotropic receptor 1 [Phyllotreta striolata]	0	66.9
IR10	252	–	N	AWT23349.1|	IR40a [Hycleus phaleratus]	8e-81	55.7
OR3	56	–	N	ALR72572.1|	odorant receptor 29 [Colaphellus bowringi]	7e-14	53.7
OR6	83	–	N	ALR72568.1|	odorant receptor OR24 [Colaphellus bowringi]	2e-32	48.9
OR11	160	–	N	ALR72561.1|	odorant receptor OR16 [Colaphellus bowringi]	6e-33	56.2
OR15	96	–	N	QNH68046.1|	odorant receptor 22 [Apriona germari]	3e-40	68.7
OR20	91	–	N	APC94316.1|	odorant receptor 9 [Pyrrhalta aenescens]	2e-11	45.1
OR23	61	–	N	AVN97860.1|	odorant receptor 48 [Anoplophora chinensis]	1e-14	51.14
OR26	267	–	N	ALR72583.1|	odorant receptor OR40 [Colaphellus bowringi]	5e-132	72.7
OR29	417	8	Y	APC94330.1|	odorant receptor 26 [Pyrrhalta aenescens]	0.0	61.3
OR39	131	–	N	ALR72550.1|	odorant receptor OR5 [Colaphellus bowringi]	2e-25	37.7
OR40	209	–	N	AVN97822.1|	odorant receptor 10 [Anoplophora chinensis]	2e-53	54.2
SNMP1a	482	1	N	ALR72542.1|	sensory neuron membrane protein SNMP1a [Colaphellus bowringi]	0	66.4
SNMP1b	534	2	Y	ALR72543.1|	sensory neuron membrane protein SNMP1b [Colaphellus bowringi]	0	54.9
SNMP2a	515	2	Y	ALR72544.1|	sensory neuron membrane protein SNMP2 [Colaphellus bowringi]	0	57.4
SNMP2b	358	1	N	ALR72545.1|	sensory neuron membrane protein SNMP3 [Colaphellus bowringi]	3e-161	62.7

Figure S1. The Venn diagrams of chemosensory genes in *P. versicolora* from the transcriptome of antennae and forelegs.

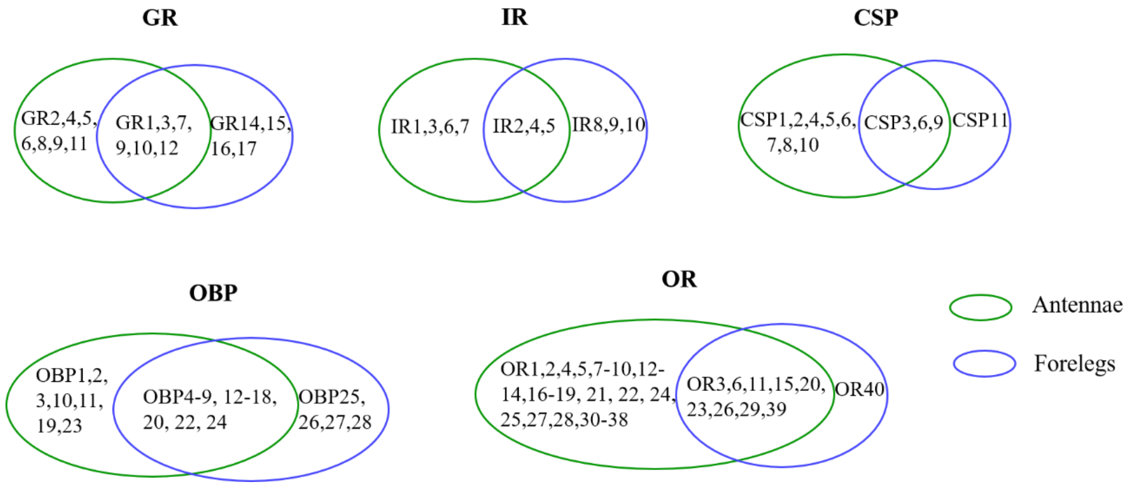

File S1. The amino acid sequences of *Plagiodera versicolora* putative chemosensory genes.

OBP

>PverOBP4

MFSFIVVGLCIICSISAFTEEEQQMMEALHAECVSQTGCPEDLISRASSGDFPEDEKLKCYMKCIFNELGVIDDDGKIDSSGLVAMFPEDIQAIAKPIFAKCGTVAGTDLCDSIYQTNKCYYGENPGAYFLP

>PverOBP5

MLKGIVIVFLSSVILDVTGMNEKQMQAAVKVVRNVCMPKTKATADDIDKMHRGDWDIEYSAKCYMSCSLNMYKLMHANNTFNYESAQKQLKTLPDTYLESTKMSIDNCRYAAETLDDKCIAAYEVAKCVYLTYPEKYFLP

>PverOBP7

MGKVLFALFLVFTVAFCKPLSDEVKAEILKIHEECAVTSGVNPREILDKVFGEGGDEDQIKTHVFCIGQKLKVIDDDNKIDRETLKTHLSEIITEDGEVEEIVTKCAVEKDDAKETAYYLTKCIHESMQKE

>PverOBP8

MNIKMGVQNEHGDIDKEALKKGLGEHNEKAQEIADECGEKKGDTAAEAALALAKCYRKHRPAPPRDHA

>PverOBP9

MKEREKIGLECLKIVNIKKEVIEEIVKTMIFPKENQKYKDFLACSYKKQGFQSQEGHILWDNITEFISRFYKNQDLKILDKCKSTSGKNHGEMAFNAMECIMNELLYMDDNIENRNE

>PverOBP12

MTLKSFLYFTVLLGLGSCIELPPELQEFVEDLHKICVSKSGITESDYAAYDVKGNPHDTKLQCYMKCLMMEAKWMNSAGAIQYDFIIDTAHPTIKDLLEPAINKCRKIDEGANLCEKASNFNFCMYEADPENWYLI

>PverOBP13

MSTVRHCLLYGVCFLVFNVRIEAAEIHKNYTNKCDIPPTAPKKIEAVINQCQDEIKLAILSEALESLNVNEHTHSRAKRAAFSDDERRIAGCLLQCVYKKMNAINDKGFPTADGLVSLYTDGISQKDYILATVDAVKYCLSFAQKKFRVTPNSIEVHGMSCDIAYDVFDCVSDEIANYCGQSP

>PverOBP14

MRGLILIFVSCYVVHSKAIECGIDKSNRDEIKQALAMCVKNNATLNKIWEMTSSAQTTPSSTEEGTDSMEENDKSVPSIPTRNIAKNQRSGKSGRIKRAKSMKSFNTQKFSTTTMRSSDDSNGMKNSDEEENNESDDVSEDKKDSSQDSTDKCIIHCVLEKMSLTDDNGLPDHSKILEELLKNTPKRELKNFLQDSTDECFQEVDEANESDSCEYSNKLIFCLAEKGRSNCADWPAGSLPF

>PverOBP15

MKPIALVAFILVLVAMIAAEDIHSRMKNIHEECQADPATKIDHGVMEKFFEGEKVDQDQFARHSLCMNKKIGLQKENGDVDKDALRKIFEHNDKLDEAVEECGQGKGTAEETALALLKCIKKYRPSRTP

>PverOBP17

MKIFLVTICVVSFVVMALVSGKDLRIKTYFHECQSDPKTYVDEELLQKGLKGELADETPVGPHAFCMNLKRGFQVPNGDVDVVALRTYLEQSESRNDTLIDRAIKECGQRNGSTAQQAALALLDCIHRIIPHKDENH

>PverOBP18

MMKTLTQLVLVATLTTVIVAYDFQNAEFNQILAEDLEDVYTNTFYHPRVRREETTSEEDKCRPRRGKPLCCGEELFRKPNDDDKDIKRACFKEITGKDKPEGRPDKHHGGPFDPFNCEKVEQFRKDMLCIEQCVGQKRNIIDAEGNLKQEDFQKYVQNQLAKDPALASITDKVVAGCLEEVKNATSLPQMDDSSCKPVGIALHHCIFKQVQLNCPEEQIKDKTACARFQERLKKGRDGMFGGPPGPGGPGGPPGPPGPPGPEGDDE

>PverOBP20

MYYNIYSIVFAIILITSMKYSSAELRHEDLGEGLLKLAELLHNTCINKTGTDDAAIDNLRNGNFVDDDKIKKYVACVWMDAQLVRPDGSMNEQLMKDLCPPKIRENGPRIVMQCWKQVKKVIPIEEKIYEMLECYYKIDPEVSTKMSECFPI

>PverOBP22
MKSIALIAFILALGAMIAADDIQERVEKMKKIHGECQADPATKVDEEDMEKFFRSETIDEAQFAKHTLCMN
KKLGLQKENGDVDKDNLRKSAERSDKVDEIVEECGQRKGTAEETALAVLKCIRKYLPKQE
>PverOBP24
MDQKVPSSKEGKCMISCFHKAVGVQNDDGSLNPNGMKVFMEKLKSNDEDMYNKFRQLLDECLIPGNVL
PDHCDTSAKVATCAIEVAKKVGLSSSFMKL
>PverOBP25
MSIIVLLFCFGVSMPQLHGATETPNQKFVRIHHSCQANPSTHIDPAELSKFDHGEDVHDPSLGSHLLCMQIG
MGLQDHDGNTPPDFQDLVKELVSEPDDERLATLAQACGKRSPGSPEELAVALRRCINEYYPELIFMHDP
>PverOBP26
MRMISQSIIIGNSILDKTKHQINSSFLRSLLFHSACLQIMVYFRLIVRYQVDFALDEVSVQVSVGSLQTLFHR
HAEHVNSNLSVVGVGSEVS
>PverOBP27
MKFPCLMLSIAAFCVPVLLIHVLCREVIRSTKSFPKSLGKNILVKNKKYIVVSPFDSLLNSESEIINFRQIHLR
PFSMLMNALSKT
>PverOBP28
MGNITVIACILAFMTLSVSCRNDRLRRFHTECQLDPAYRVEEGLIRKAMRGETVDESVVGPHVLCINIKAGI
QNQNGNVIEKGLRTILKERIKDDSVVEDVMSRCGHKNGGTPHEAAVALTRCLHKHARNH

CSP
>PverCSP3
MGVCTIFLICLVGLVLAKPDEKKYTTKYDNLDIDTILKSERLLKNYVHCLLDKGKCTPDGAELKEHLPDA
LLTDCSKCSEFQKKNSKKIIRYLIDNKPDWYKELEAKYDPEGTYKAKYDEEIKDKAAE
>PverCSP6
MHLFSIFIFVFLVVSAIGEKYTTKYDNIDLDEILKSERLLKNYIDCLKDKGNCTPDGKELKETLPDAMRTEC
SKCSDKQKEGSKKVLKFLLKNKRATFDELASKYDPEGYYVKRYKDELAKEGIII
>PverCSP9
MKSIIVFCFVVVLASVIARPDDSKYTSKYDNVDLDAIIHNDRLLRNYVDCLLGKKKCTKDGEELKRILPDA
LKTKCAKCSEFQKNNAKKVINHLIKNQRAWWDELEAVYDSEGIYRKEYEAEAKKEGIDLN
>PverCSP11
MFRLSFLFCVLVVVSLVEGQKYQSKYDNIDVDSILTNRRVLKSYLRCILDEGPCPPPAREFRTHIPDAISNNC
ARCTDAQVNIIKKTSKFIMRNNPEDWEKISKKFDPQRKYRASFTKFLNA

OR
>PverOR3
MIIVGHVFKTMRERTLRRLDFPEKYALMHDSDNPALEKELYSQLNKAIEHLMVLLR
>PverOR6
MDCHSGQRIQDESDSIAMGLYQANWDQCDSELKKDILFVLMRCQKQMILKAASFGVMDHPMFLAVNCH
IHNIKYLFPVRISDL
>PverOR11
MYHIYSFMSTNTATYFYCGRIFLDGLPVDRNLPFESHIPFDIPFTVLFVYELIIGKSLKEGIRAFSYSATLFFEF
FMCYCLPAQYLNDEAELLANSIYFSNWHLHSKHSKDLVLLLGKSHLKMEMSAGGYTNIDLKAGFAALKS
MVSYCMFLRTVGVSQD
>PverOR15

MNDAFGVMMLIHITWTSFIISVLGFGIIMENNYWNMMRFIMHLGGWLLMLFLVSFYGQILMDKSSDIAKE
VYDSEWYWTSPKNRESIVLILLRSQR
>PverOR20
MNAMLVECVDQHRRISAFKSEVSSIFGYLTLTQFVISVLTLCTNLFELTMVTELNAQLFFLVFYLIFLLIQIFF
VCYFGNEIISKNFQLTN
>PverOR23
MTEWYTADIKIRKYLVIFMERVKKPIIIRAGYFFPLNVETLASILRSSYSAFTVLQQFYKD
>PverOR26
MLKSLDKLERTFLIYISCSTCMFLFKPLLVRGSTIYYYYQIPQIPFLLSYGVEFYVTLVTMSMVIAVNIFISISI
VIGAGQFSNLNARIRQMDVGKAAEDGGMENVMGVLKRNVAYHNYLIEYVDRLDDIFSLLFMVLVEIITAL
LCMNMYVLSLPNTTVVDYIRCGTMVCAFTTEFLFLYGVPAQKLMDEAEQVANSAFYHCDWYLPNILPIR
KSLTFMIHRSQKTVGLSAMGFIDINRQTIVAMIKTAYSFFTFLQTVESSDTPK
>PverOR29
MSDNYADDFFVVNRWILRWASVWKPENGNKNIQTLYMIYAVVVFVLVDIYFIGTEFLSLISKFGNEYEFMK
ICSFLLTHLMGASKVLFFHFRGVDLRKIMSTLESAQFRYGSCPEKGFFPGETSKHFKRVGIMCSMLFLLMA
HVTLMSSYVPSLVAALQHVEGNPEKPLPDRMPYYSWMPFRFDTTLLYVIALVYQATLLFFQAHCIVGMDT
LFMNIMNCIGMNLEIIQGAFFTVRERASEKVNGPLLTTDGLYNSEELKTALNDEMRKIIKHLQTIYKMCDD
LEDLYKFLTLAQTIATLFILCSCLYLVSTVPATSEQFLMSEVVYMIAMGFQLILYCWFGNEVTLKADELPFHI
WQCDWISADTKFKKSIILTMARAKRPLYLTAGKFAPLTLATFVSIVKASYSFCTVIKKSSH
>PverOR39
MSVLTLFVDEDVLVYECYRPTWLPFYVLWLYQISAVNMAMLIPIICFDTLLMNFINLTYLQFRLLNRKIEAI
MESGDVLEIRKKIRLVVEHHHFLMKYTQLINISFSSCLLAYMIMIVTSMCVEMYNISSK
>PverOR40
MVIKAIPLSAWYPYDEQKHYLPSFCWNVLDGSIGASFLAYSDILSFSLIIFPLGQIHILKHIVANFDDYVKKIE
NQIGVSQKEASFITIRECILNHNEIIRYIRDLNDAMRNVMLLDFLQSSIQLASLVLQLFEDLILPNIVLFGQFL
CSMLIRLLLYYWYSNEITYQVFLNFFKSNTIMVWCFLNFYFRALTLQLQYGTANGMNNLRK

GR
>PverGR1
MWTKFQYKYFIVTGTPIIFRNLSVITYTLCTVSWLAGIVLMLAQYYLQSDMLLWHTFAYYHILAMLNCLC
TLWFINCTAKGRAAGWLAENLHNALQSSDPAVRLAEYRDLWVDLSHMMQ
>PverGR3
MGDFLIIALSLIVSSRLKQVSMGLESSLRNKRFTSSISTNQETRGRFYTRIREDYQRIAAICERVNELLSMIIIN
SYVANIFFLLVQLYNSLKPMGSVIERFSYFFSFAVMIFRLVLISIYAASVNEESKRFLLVLNNVPSELWHHEVE
RMIGQIDFGGAQLSGYGLFTIDRGLILKVCMTVLSYELILIQFGNSSTADGGSFQNVSESNITIIDY
>PverGR7
MFRTLEISTSAIGVSYVFWSSAYLSLICRNDWTRLLSKMQQLEHEMPASKAGGSHHALRARVVFVYSFLFS
FHTTRECVWYSNQAYNHIGAFSFTFITHFYLTSLLVQHVVFSDWLANRYDTLNEELARWGRTPHRLGAEL
EDFTKLHKLAGALVVAHNRIFDSLNFVQVSVYVVFILNTLAYSVDGHQSTMGRKILDLVQPLFHSVSLLLF
TEACDAVEKSGRRIVRTCYSLHKNIQGDALLKEKVKVLARYAEERRPVFCFWNFFHLNRSSLLTFFSAIITY
TVIVIQFNLSVKKKIVSNSR
>PverGR9
MLKTVVLSRENFQFFQPILTVSRIFGLCPVSCKYDGPRVIIGWSNLLAIYSYLLSIHFIIVSIIGLVNDIDEGDS
KSFRMKMRKGKFITCCDVIIIILIVIFGTITIPCRMRRFMDILKNFNKTDSVLGLPNLKGYRRQSIRMILAVYC

LFTSVVVFDIFYWTKSSCRHEKTGGQYILRYIGFYLLYYIMITLELYFSHLVYSIHIKLALINEHLVIGNTVNN
SERDVDVISSRIDDFHHQICRCTGPKDFETIFKIKIPRTRINNAQRIEILRNLYKQLNNGVKIINETIANGIMLI
MLSCLVHLVVTPYFLVAELIKDKSNYFFIFLQVLWIITHTCRLLIIVEPCQACSLEGQRTTMLLCDLLGLSST
DESFRKAVQSFSIYLSQNQIKFSCSGLFTIDRSVITSVAGSVTTYIVILFQFNNN

>PverGR10
MESTVQKAYFYISFAFLLVRVLGVCVLGGAVHEEWGNIRFHLGHVGSLAYNEEVERMVHHVTTWELSLS
GKNFFFISRELILSMAAAIVTHTLVLIQFYS

>PverGR12
MVLFIFDIIMWYHRGNNNSTYLKEYSTFYFLYAMVVMKEVFFWHVIFLIKIKISLLNNKLLDIKNKAISTDI
HFIGLGGNLNTIDNGYKCGNSKDLGGYFLLTKIH

>PverGR14
MTYLLIYAQRVFKMLQENVFVFLWLLLAIEVYKRFVFLNNSLVRVFEDAQRDVTFPRKSLVMEKFKSKDD
PMEQVRKIGEMHNHLCELIDEINQIFGTGILFFTLFCIMFVVSCTTVTIQSVFIETGTELQLGRDSNYSNCSFI
VLTFVVVIFLSAAGDYVSGEAKRTKDICQNIVVGLNMYDHSKDDLNQLAQQIINRNPQLAASSFFAIDFTV
LGFMLANITSYIIVAVQFLK

>PverGR15
MYLNTISLLEGSGPENRRRRSVYLEGAQPFYQNARRDPNPVTKVAPFQPSGSGNPTKLFLDQKQQRSSLFD
VLKPIVFLMRAIGVFPIGNVGDVFQVTAQMLAYSIVVFLVVLTYIAYIRWDKVEMFRTAEGRFEEAVIDYLF
TVYLVPIVINPIAWYEGRKQARVLTTMMSFEKIYRRVTRKNLSVSLGNTPLVITIGLPALAISAMVVTHVTM
VHFKLFQVIPYCYINTTTYLIGGSWYIYCDLIGNIAQTVAADFQQALKNIGPSSRIADYRALWMMLAKLVR
DVGNAFAYAVTFLCLYLFLIITLTIYGLLSRIQDGVGIKDVGLTLTAFLSGVLLFFVCDEAHYASNCVKVQFQ
KKLLLVELNWMNDDAQQEINMFLRATEMNPTDMSLGGFFDVNRNLFKSLIATMVTYLVVLLQFQISIPGD
DYSEGDASKTNATKIK

>PverGR16
MVLVQLAYPVFFIGYMISLTMNFHYIKGIGEKISKTCFTLKWDIDKQVRKELIFLNRFMWHVRPKISAAGFF
SVNQDTLSRMFNNMFTYFIVCVQFDSTFL

>PverGR17
MYIVYLSYLTMFVLFINRPILGTPSPKKIGFVNYVSIVYVISFFVKLVIIAAAGDLATQEARKTTKISYNILTK
LGPDIKDRILKDKLLALVQQVAVRNPQFFATSIFTLDFKILGSIVRQITSYLIVIVQVMSVMKLQ

IR

>PverIR2
MPVNSIIYKVGNTKSSRIVVEALYKFDRTSTNFLTNRIAEWSPENGFRYFRKLISTTNRTNFMGTPIRTSYVIF
DNDSLNHLSDYRFIDTDKLSKINYRLIDSLKDLLNTSRVNIWTSSWGNKNLTTGEYSDGIFKDLLSNRADIT
GTTAFISKDRLDSFTYLVPTTYEEIKFVFRAPSLSYTKNVYTTSFSPMFYLSCLSILVLGGFLLYFLELVEYQC
GAIPNRMTLMDVVATQICSITQNSVIRMPELPATRIAVFFNMIFCMLVYTAFSAYIVILLQKTTDEIKDVRSLY
DSKMPIGVQNTPYNKYYFSTPNLLTNEHYRKLFYENRLGGSQSNFISPEKGMKNVQGGFYAFQVEQATAH
YFIKTTFNENEKCSVKSIPTIFVGFQPYLVIPKNSSYYNHFQVGFRRLFETGLHQREYVRFFTKEHKCQGAQ
RNYDSVRLMDCYFPFLIFAFGFTLSIFVLMTEKIIKRIGKKGSMQEVCRTKDGYRKRIAQPLDFIN

>PverIR4
MQNTEKIDFVLVDLVDDDAVNKEKICETLSDGGTILLDLTWFGNDIGRTHAYDIGVPYIKIDVTISPLLDILD
KYLDYRNSSDVVIIFENPSYIDQALYHWINTARMRMLISETLDAETAKKLKGVRPTPSNFALVANTKNMQR
FFEVAIRENLVELPERWNLIFLDFNYKSFDQSLMKNQPINYLSLDTNICCKIQSLPNSCECSDTFMMQKEFL
RAALKVVSTSAQEMMRNGLLDSSLACNNNDSSVKDDIDTQFQDILKRELSGQNVMYLEKSIMRMITSGFI

EIGSDSNTNTVAKYESGVIRPEKNATIKPIKAFYRVGITHALPWSFKTIDPDSGALVWTGYCVDFTKKMAE
MMNFDFEFVEPKSGTFGEKINGVWNGVVGDLAYGQTDLAMTAIIMTADKEEVIDFVAPYFEQSGITIVMR
KPVRKTSLFKFMTVLKLEVWLSIVAALIVTGFMVWFLDKYSPYSSRNNKNAYPYPCREFTLKESFWFALTS
FTPQGGGEAPKSLSGRTLVAAYWLFVVLMLATFTANLAAFLTVERMQAPVQSLEQLARQSRINYTVVKES
ETHQYFINMKNAEDTLYRMWKELTLNASTDDTRYRVWDYPIREQYGHILLAINDSNPVADAEEGFRNVE
DHLDADYAFIHDSSEVKYEISRNCNLTEVGEVFAEKPYAVAVQQGSHLQDDISKTILTLQKDRFFEGLHAKY
WNHSSKGNCPNIDDNEGITLESLGGVFIATLFGLALAMITLAGEVIYYRQKRKTIDFKKKQAKGQLPEKYF
KQNRMITIGTSFQPTQFNQKAVQDQKELKLSHISLYPRARNRITQVE

>PverIR5

MKMGLNKIWLVFLSFLLENCQGETTQNINVLFVNEENNGVAEKALDVAMTYLKKNNKLGIAVDLKKVV
GNRTDSNKFLEALCSTYNSMLSTQTFPHLVLDMTMTGLGSETVKSFTQALALPTISASFGQEGDLRQWRN
INESETDFLIQISPPADVIPEIIRTIVLNQNITNAAILYDSSFVMDHKYKALLQNVATRHIITPIKEVSQLAEQLT
QLRKLDLVNYFVLGNLKSIKNVLDAADGLNYFNRKFAWHAITQDDGDIRCTCRNATILFAKPLPNALYQD
RLGAMRRTYQLNAEPIVASAFYFDLILHALMAVNEMISDGSWKSGGGGFITCDEYDGSNTPKRGGLELRR
FFSKQSSEDPTYGPFSVASNGWSHMEFQMQLTAVGVRDGASDKSVNIGAWWAGFDNNLTLLDAQAMGN
LTADVVYRVVTVEQKPFVFRDESSRSGFNGYCVDLIDKIAEILKFDYEIVAVDNFGVMDENGKWNGVVKE
LVEKRADIGLGSMSVMAERENVIDFTVPYYDLVGITILMKLPESPTSLFKFLTVLENEVWLCILAAYFFTSF
LMWIFDRWSPYSYQNNREKYKDDEEKREFNLKECLWFCMTSLTPQGGGEAPKNLSGRLVAATWWLFGFI
IIASYTANLAAFLTVSRLDTPIESLDDLSKQYKIQYAPLNSSSTQTYFERMANIESRFYEIWKDMSLNDSLSE
VERAKLAVWDYPVSDKYTKMWQAMKEAGLPANMAEAVERVRASKSSSEGFAFLGDATDIRYLELTNCD
LTRVGEEFSRKPYAIAVQQGSPLKDQFNTA

>PverIR8

MTTISAEEVHHLPTMRLGAALLVTTLATLLVESHLLESIQEAKWADSFLKGTKIESTLHIVELLKYIALNYL
TDCTPMILFDSSEKNDDLFIEKLLTQFPIPYFHGRISDSYEMMMGSDSAPTTCVSYILFLTDVMRCRNIIGDI
SHQRVVVIVRSSQWRVLDFLMHEDSRVFMNILVIVKSERIVSPQKPIFQEAPYILYTHELYADALGSSRPIVL
TSYQHGRFTRLVNLFPKKMSTGFSGHRFVVAVAHQPPFVSR

>PverIR9

MKRIIQSVVLILFCFGFQMVSSSEVTIGVLLNDLTSQVQLPLNSVIYNKNVFDQNAHFSTDVSLVSNIDSFD
ASRTLCNMMNSSIGITAVIVEDIPNTIPVLESICTNFEIPFIMTSWRPPVVRNPDQERALLSFYPEAERFAEGL
AEIVKSLQWTSFVIVYENEDGLIKMQDVLKLQEYKKNTRRNNIFVKQLGPGPDYRPLLKEIRNTTEDNMIL
DCKTENILPILLQAKSVNMLNLHNRILITSLNAHTVDFSVLNTTANITILRLHDPKTENFENAIHRWQLTEFE
NRDIHTQLDPKSIMTETLLFHDAILLLTDAVRDLSITPKIETSPISCSENQTTRDGFAIRNYMRIKTPSMTLTG
PLEFNNNGDRIKFNIYAIDIIEDTVIATYFAGNKSITLARSGKESMDAAVLNLQKITVIVSSRLGPPYLMPREP
TYEGEEFVGNRRYVGYSMDLIDGIAKIIGFKYEFVITSKYGKYDEEAKRWNGLIGELLEKRAHLAVCDLTI
TPERTEVVDFSMPFMTLGISILYKKPDKKDINMFGFLETFSKAVWIYTATLYLIISIVLFFISRMTPGDWENPH
PCEDEPEELENIWDIKNCLWLTLGSIMTQGCDILPKGISSRMAVAMWWFFSLIMTSSYTANLAAFLTKANL
EPEIDGAEALSKQTKIKYGFLAGGSTESFFRNSNFSVYQRMWLNMQQFKPSVFEENNADGVNRVQTTKN
SLYAFLMESTQIEYEVETKCTLKQIGNWLDTKSYGIAMPMNSPYRTAINRAVLKMQEAGELGLLKKKWW
KDERKEPSCDQNASDDGDSAKLALANVGGVFLVLGVGIAMACIFAILEFLWNVRNITVEEHVSYWEALK
VELIFACKVWITKKRTKRLMTESSSSSSEKSDRTDDRSIIHSILHSAGSFMHLNAQS

>PverIR10

MRTRNVKLFVEQHSSYYGLLENGTGIYTKLWDLMVNKQGGDFLIKSVEEGVKLVRGDREIAVMAGRETL
FFDIQRFGASNFHLSEKLNTAYSAIALQLGCPYTEEINKILTAIFEAGIITKMTENEYEKLGKQKELTSDIAES
VKKEANKESRLSKKDNEENEKLKPINLKMLQGSFYLLCFGHVFSGLILLGEIFLHKHYIRYNSRKKRFKMA

RKLMRNLSLKTSRIRLAIRTVYRNLMHEAFVSTLEYIE

SNMP

>PverSNMP1a

MLQMINLGPGTDIRDMFLKVPFPLTYRVYIFNVTNPDRIQKGDMPVVNEVGPFCYEEWKEKMNVEDMEA
DDTIAYDPKDTFLKKRWPGCKTGKEIITVPHPMILGLVNTVARQKPGALSLANKAIKSIYANPSSIFITTEAD
NILFDGVIINCGVTDFAGKAICSQLKSSGNLKLINGDQLLFSLLGPKNATLNTRMKAYRGKKHFQDVGRIV
EFGGAKNLDVWPTDECNEIKGTDGTIFPPFLKKEQGLVSYSPDLCRSLRATFVKDTVYDGIPCAEFTATLG
DMSKNEDEKCYCLTPDTCMKKGIMDLYKCAGVPVYASLPHFYGTDKSYLDGVNGLTPNKSKHEIKILFES
TTGSPLYARKRIQLSMPLEPIQKVELFMNFTPTVIPVLWIEEGVELNRTYTGQLKSLFTMKKIVGAFKWIVL
LSSLGGLAAAGYMFYKNNGKIEITPIHESKRDGISTIHSLEGQVNHGMSENSIDKF

>PverSNMP2a

MLACSRFFSNKVLAVLTTILTLIFVGVLVLAFYGIPVIINKSIHNSVHLEKGTIQWDRFVDLPVDILMKVFLY
HVTNPDDVLNGAKPIVEERGPYCYKQNIHKNILSTSSSQDTVTYEQNFKIEFDQEASGNLKESDKVVIVNP
VMLTLYKLTSRLERLVVFGCLDKIFPKEYIGVFIEVDVKTVMFDGFAFAQRSEDLGPACNIVRNQILDKTLP
MKNVERITDDDGILELRFALLQYKIRGPDGNYTINRGIDDITKLGHIIKWNDETELPFWGRMQSINNDTCK
KVRGSDSTIYPPQVDKTRSFDIFSTDICRVVEISFQRTDTYNGIDAYRFGITKNTFRSATTNPENDCYCIKQS
AGIDGEPSCYLDGVLDVYPCFGAPILLSFPHFLYADESYVDAIEGIGPSDPDIHELFLLIEPNTGTPLQGMKR
VQLNTVLMPMQNIPGTSKISPLVMPILWLEEGVSLPQNLIDELNSHYFQTVKLVEGIIYGLIAVVAASVLISS
GFLIRRKCC

>PverSNMP1b

MQLAIKVLVSGVGITISSVIFALVIYDPLIKYVIRDQTSLKKNNEIRDIYLKIPFPLDFRIYLFNVSNPMEVQD
GAKPVLKEVGPYCYDEYEEKVDVIDNEMEDSLTYNSYDIFRFNANKSIGLSENDYVTIIHPLIVAMAYQVN
RDTPALLSFLNQAIVTIFKNPKSIYLTDTVKNILFDGFEINCNVTEFAAKAVCTQIMNSNIPGLKTDPSRNNT
LVFSLFGARNATLGHTMKVLRGIKRSEFVGKVLEVDGKKEMNLWTSKACNRYRGTDGWIIPPLLEPGVG
VWTHSVDMCRNVEAKYIKETVLNGVNARLYEADLGDMQKNEDEKCYCPTPSTCSRKGTFDLTKCMGAP
IIASLPHFLRADEIYRQQVDGMQPVHEKHIISIYLEGVTSAPLRATKRMQLNFPITTVPKLTLMTKLPEALHP
LLWLEEGVEVEGEFLKLITDKLMLLNVANYGRWLAVFGGLITTGVGVYLHNKNKNSVAISTIHSGDIDREI
TRSTNELMDQMNRIQGNEKGHVNHVLSGHEFDRYM

>PverSNMP2b

MLLKVADIQAAALPMVEGVLGDIFKENDGLFIKVKVKDYLFQGLKMCENEGKDGDFAAGLVCKQVIAEA
ATSNNLRVENNTILFANLHYKNNTHLGRFTIKAGIQNHNEIAHLALYNNQSYISIWGEEKSICNKIEGLSTTV
FPVNINKDMIFESFAEDICRRMKLTYKMDETVKGLKGYKFTAANDSFSMKNENNTCYCNKKTTLMDGKL
GCVKDGITDLSTCTGGPVMVSFPHLLYADKEYLNSVEGLDPDSMKHESFVVLEPMSGFPLSLAQRVQFNIF
LRPIDESTILANVSRALFPLIWVEESLQLDDKFTDMLKNNLFKTLDMINILKWVVIASGSACFLFAVSMAIY
NDAS
